# DNA Vaccines—How Far From Clinical Use?

**DOI:** 10.3390/ijms19113605

**Published:** 2018-11-15

**Authors:** Dominika Hobernik, Matthias Bros

**Affiliations:** Department of Dermatology, University Medical Center, 55131 Mainz, Germany; dhoberni@uni-mainz.de

**Keywords:** DNA vaccine, nano carrier, promotor, transgene, adjuvant, antigen presenting cells, dendritic cell, macrophage

## Abstract

Two decades ago successful transfection of antigen presenting cells (APC) in vivo was demonstrated which resulted in the induction of primary adaptive immune responses. Due to the good biocompatibility of plasmid DNA, their cost-efficient production and long shelf life, many researchers aimed to develop DNA vaccine-based immunotherapeutic strategies for treatment of infections and cancer, but also autoimmune diseases and allergies. This review aims to summarize our current knowledge on the course of action of DNA vaccines, and which factors are responsible for the poor immunogenicity in human so far. Important optimization steps that improve DNA transfection efficiency comprise the introduction of DNA-complexing nano-carriers aimed to prevent extracellular DNA degradation, enabling APC targeting, and enhanced endo/lysosomal escape of DNA. Attachment of virus-derived nuclear localization sequences facilitates nuclear entry of DNA. Improvements in DNA vaccine design include the use of APC-specific promotors for transcriptional targeting, the arrangement of multiple antigen sequences, the co-delivery of molecular adjuvants to prevent tolerance induction, and strategies to circumvent potential inhibitory effects of the vector backbone. Successful clinical use of DNA vaccines may require combined employment of all of these parameters, and combination treatment with additional drugs.

## 1. Introduction

Compared to conventional protein/peptide-based vaccines intended to induce antigen-specific adaptive immune responses, DNA vaccines are more stable, cost-efficient, easy to manufacture and safe in handling [1]. DNA vaccines are being investigated for various applications including therapy of cancer [2], allergies [3], autoimmune [4] and infectious diseases [5]. In the US, at the moment over 500 clinical trials that focus on DNA vaccination are registered, targeting especially viral infections [6] and cancer [7], while bacterial infections and autoimmune diseases are less of a topic. The high number of DNA-vaccines tested in clinical trials emphasizes their important role for future medical approaches. This review aims to summarize current achievements and ongoing developments in the design of optimized DNA vaccines and delivery methods.

## 2. Course of Action of DNA Vaccines

**Figure 1:**
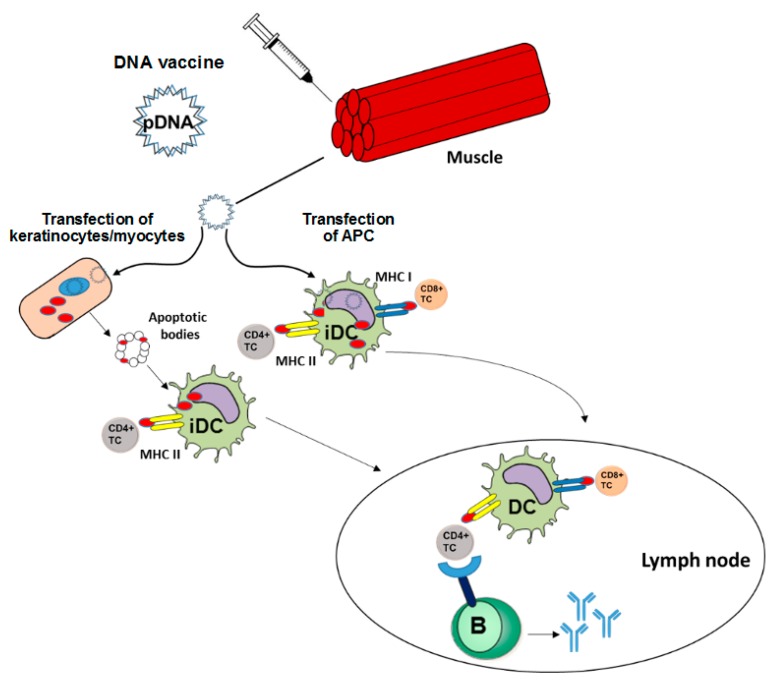
DNA vaccines induce adaptive immune responses. A DNA vaccine intended to induce an adaptive immune response needs to encode an antigen and an adjuvant. The according plasmid DNA is applied either systemically or topically, e.g., by intramuscular injection. Transfected keratinocytes or myocytes express transgene and release derived peptide/protein via exosomes or apoptotic bodies. This material is endocytosed by immature dendritic cells (iDC) which subsequently present antigen preferentially via major histocompatibility class (MHCII) to CD4^+^ T cells in draining lymph nodes. Direct transfection of APC including iDC results in endogenous transgene expression, and hence parallel presentation via MHCI and MHCII, yielding CD8^+^ and CD4^+^ T cell responses in parallel. Besides this cellular immune response, a humoral immune response is induced if the B cell receptor recognizes the protein antigen, and acquires help by pre-activated antigen-specific CD4^+^ T cells.

The classical ways for vaccine delivery are intramuscular, intradermal and subcutaneous injections which address primarily myocytes [8] and keratinocytes [9], respectively, but also antigen presenting cells (APC) residing near the injection side [10] (Figure 1). In case of DNA vaccines, after their internalization the DNA needs to translocate to the nucleus for transcription, followed by translation in the cytoplasm [11].

APC can be transfected directly by DNA vaccines [10]. In this case, the encoded antigen is expressed and after processing antigen-derived peptides are loaded in parallel onto MHCI and MHCII molecules [12]. In case of concomitant activation, antigen-presenting APC migrate into the draining lymph node and can prime CD8^+^ and CD4^+^ T helper cells [13].

In case of cutaneous application, transfected keratinocytes may generate antigen which is released by exosomes or apoptotic bodies and is internalized by APC [14]. In general, antigens of exogenous origin are loaded rather exclusively on MHCII resulting in activation of helper CD4^+^ T cells which in turn contribute to B cell priming to yield a humoral immune response and are required for full activation of CD8^+^ T cells [15]. So far, only subpopulations of dendritic cells (DC) with so-called cross priming potential are able to load MHCI with internalized antigen [16].

After intramuscular application, transfected myocytes may undergo apoptosis. Apoptotic bodies are engulfed by APC and the exogenous antigen is cross-presented on MHCI resulting in a CD8^+^ T cell response [17]. Early studies on DNA vaccination demonstrated that priming of CD8^+^ cytotoxic T lymphocytes (CTL) is mostly dependent on bone marrow-derived DC rather than induced by tissue specific cells [18], and was strictly dependent on CD4^+^ T cell help [19].

The most serious challenges for DNA vaccines intended to induce an anti-tumor immune response are caused by immune evasion strategies of the tumor [20]. In this regard, tumor cells are often characterized by defective processing of antigens for subsequent presentation via MHCI [21], and impaired expression of MHCI [22]. In addition, within the tumor microenvironment both parenchymal and infiltrated immune cells may overexpress tolerance-promoting non-classical MHCI molecules like human leukocyte antigen (HLA-G) and HLA-E [23]. Besides, tumor-associated macrophages (TAM) protect the tumor from immune responses by various mechanisms including the release of anti-inflammatory interleukin-10 and the expression of surface receptors like programmed death-ligand 1 which inhibit the functions of activated T cells [24]. The effectiveness of DNA vaccines can also be diminished by CD4^+^CD25^+^FoxP3^+^ regulatory T cells (Treg) that promote cancer growth which inhibit T effector cells [25]. Moreover, both TAM- and tumor-derived mediators promote the expansion of myeloid-derived suppressor cells (MDSC) which interfere with the activation of T cells in the tumor microenvironment and attenuate T effector cells as well [26].

## 3. DNA Vaccines in the Clinic

The use of DNA vaccines has early raised safety concerns mainly concerning the probability of stable integration of transfected DNA into the genome of somatic or even germ cells, causing dysregulated gene expression and mutations. In this regard, Wolff and collegues who demonstrated that a luciferase-encoding vector after intramuscular administration was detectable for more than 19 months in skeletal muscle, but only as an extrachromosomal plasmid [27]. However, Wang and coworkers reported that intramuscular injection followed by elecotroporation strongly enhanced the overall transfection rate, which was associated with a low level chromosomal integration of vector DNA at random sites [28]. However, the authors calculated that the integration frequency was well below the number of spontaneous gene mutations. In a subsequent study, the majority of plasmid DNA administered into skeletal muscles of different rodents was found to remain at the injection site, while minor fractions were also detected in other organs, including the gonads, but not integrated into the genome [29]. Concerning unwanted DNA vaccination-associated immune effects, repeated intramuscular application of a luciferase-encoding reporter vector in primates resulted in long term reporter expression, but induced no anti-DNA antibodies [30]. Potential transfer of prokaryotic elements of DNA vaccines like antibiotic resistance genes into e.g., the gut microbiome has been considered another issue of safety concerns, but so far no such event has been documented [31]. Nonetheless, the aforementioned as well as additional safety concerns of DNA vaccines need to be considered with regard to their translational use in the clinic [32].

Presently, there are no approved DNA vaccines for use in humans. Nevertheless, some DNA-based vaccines were approved by the FDA and the USDA for veterinary use, including a vaccine against West Nile Virus in horses [33] and canine melanoma [34]. One of the first human clinical trials with DNA vaccines evaluated the therapeutic and prophylactic effects against HIV in which no significant immune responses but a potential immunogenicity were detected [35]. However, in the same year Wang and colleagues demonstrated induction of CD8^+^ T cell responses in primates after immunization with a mixture of for plasmids encoding for different Plasmodium falciparum proteins [36]. Another clinical trial which targeted the Hepatitis B virus showed the induction of a humoral response in patients not responding to conventional vaccination [37]. The overall safety of DNA vaccines has been thoroughly proven in several clinical trials, underlined by the fact that no antibody response against prokaryotic parts of the DNA vaccine itself has been observed and that adverse effects are limited to mild local reactivity at the injection site [2].

One of the first clinical trials employing a DNA vaccine for tumor therapy was conducted in 1998 using prostate membrane antigen as a prostate cancer antigen delivered using an adenoviral vector and granulocyte-macrophage colony–stimulating factor as an adjuvant. In this trial delayed-type I hypersensitivity responses were observed [38]. Therefore, subsequent trials pursued the goal to improve this parameter [39]. Vaccination of melanoma patients with a bivalent DNA encoding Melan-A and tyrosinase as tumor-associated antigens elicited humoral and CTL responses in Stage IV patients [40]. A subsequent clinical trial used the Synchrovax vaccine encoding for four peptide epitopes of Melan-A and melanoma antigen recognized by T cells 1 to induce broad antigen-specific CD8^+^ T cell responses [41]. This trial showed antigen-specific responses but no induction of tumor regression in the patients. Recent clinical trials showed good results in inducing immune responses against Wilms’ tumor antigen 1 using DNA vaccines encoding for two different CD8^+^ T cell epitopes [42].

Altogether, clinical trials employing DNA vaccines evoked efficient induction of cellular and humoral responses [2]. However, the level of these responses most often was not sufficient to elicit significant clinical benefits. Therefore, numerous clinical trials focus on DNA vaccine optimization strategies to augment theirimmunogenicity [7] as presented in the following. In addition, it is necessary to compare the suitability of different DNA vaccine delivery routes to yield potent adaptive immune responses. In this regard, the Cutaneous and Mucosal HIV Vaccination (CUTHIVAC) trial is worth to be mentioned since it aimed to comparatively analyze the influence of the combination of different injection sites with or without electroporation [43]. In the following optimization strategies for the design of DNA vaccines and approaches to improve their delivery to APC are presented. Both aspects are important to overcome the poor immunogenicity of DNA vaccines in human.

## 4. Optimization of DNA Vaccines

**Figure 2:**
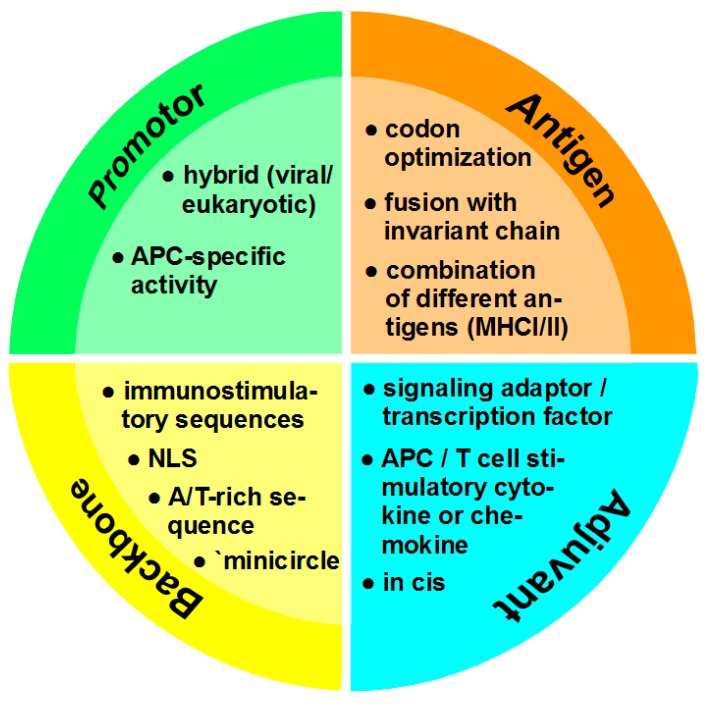
Optimization parameters of DNA vaccine design. Use of a hybrid viral/eukaryotic promoter was shown to prevent transcriptional silencing as observed for viral promotors. Alternatively, a promoter engineered to transcriptinally target APC like DC may be used. To enhance antigen expression, especially in case of pathogens, codon optimization is important. To induce a broad CD4^+^/CD8^+^ T cell response, linker-separated sequences encoding different antigens may be used. In addition, fusion with a sequence encoding the invariant chain may enhance loading of antigen onto MHCII. Conventionally, molecular adjuvants intended to enhance the APC activation state and/or T cell attraction and polarization are encoded by expression vectors coadministered with a DNA vaccine. To ensure coexpression of antigen and a molecular adjuvant by a transfected cell, both sequences must be incorporated in the DNA vaccine (in cis), separated by an IRES or T2A sequence. The vector backbone comprises the part of the DNA vaccine which is not required for eukaryotic expression. Inherent or inserted immunostimulatory sequences are detected by danger receptors, and mediate APC activation. Inclusion of a NLS facilitates nuclear entry of the DNA. Intrinsic inhibitory effects of the backbone on the transfection efficiency are limited by insertion of A/T-rich sequences or by recombinase-mediated deletion of the prokaryotic part as a last step after propagation in bacteria to yield minicircle DNA.

DNA vaccines still face many challenges to become an effective tool as their success achieved in preclinical studies has not been translated into the clinic yet [44]. The biggest challenge is the low immunogenicity of DNA vaccines in bigger animals and humans probably due to the difficulty to upscale the DNA vaccine amounts used in small animal systems [45]. For this, about 5–20 mg of DNA would have to be injected into an average-sized human [46]. If naked i.e., unformulated plasmid DNA is used for vaccination, an important factor that contributes to low therapeutic efficiency is DNA degradation [47]. Studies showed that plasmids, compared to other administration forms like minicircles, are degraded relatively fast within one week [48]. DNA molecules that successfully enter the cell need to pass the barrier of the nuclear membrane to be transcribed [11]. Hence, due to the low amount of DNA that is at disposal for transfection of a given target cell in vivo, a major goal is the optimization of transfection. Optimization parameters of DNA vaccine design are summarized in Figure 2.

### 4.1. Promotor

Conventionally, viral promoters like the human immediate/early CMV (cytomegalovirus) promoter which are ubiquitously active at high level have been employed to drive transgene expression [49]. However, especially with regard to long term expression, viral promotors are often subjected to methylation-mediated inactivation, whereas eukaryotic promoters and hybrids of eukaryotic/viral promoters remain active [50]. Furthermore, the use of cell type-specific promotors may allow to restrict antigen expression to APC.

To achieve transcriptional targeting of DC which at activated state constitute the most potent APC population [13,51], various promoters of endogenous genes that are expressed predominantly by this APC population were tested to drive DC-focused transgene expression. CD11c has been well established as a DC-specific marker in mouse [52], whereas this ß2 integrin is expressed more broadly in human by different immune cell types [53]. Transgenic mice engineered to express antigens under transcriptional control of a large 5.5 kb promoter fragment showed DC-specific transgene expression [54]. However, when mice were biolistically transfected with a DNA vaccine containing this CD11c promoter fragment insufficient CTL activation was observed [55]. Dendrocyte expressed seven transmembrane protein (DC-STAMP) constitutes an evolutionarily highly conserved transmembrane protein of the endoplasmic reticulum [56]. In the same study, DC-STAMP was reported as primarily expressed in immature human and mouse DC, down-regulated in response to maturation. However, osteoclasts and macrophages were reported to express DC-STAMP as well [57]. The suitability of the DC-STAMP promotor to transcriptionally target DC was evaluated by transduction of hematopoietic stem cells with a lentiviral expression vector harboring the DC-STAMP promoter to drive expression of a fluorescence reporter [58]. Lethally irradiated mice were reconstituted with transductants, and reporter expression was monitored in different immune cell types. Indeed, the reporter was primarily expressed by differentiated conventional and plasmacytoid DC, and at low level in monocytes, B cells, and NK cells. Dectin-2 (CLEC6A) is a C-type lectin receptor (CLR) that binds mannose-rich surface structures of various pathogens [59], and is primarily expressed by Langerhans cells (LC) [60] which constitute the epidermal DC population. In line, biolistic transfection of mice with a reporter plasmid driving luciferase expression by the Dectin-2 promoter yielded predominant reporter expression in LC [61]. In subsequent studies, transduction of mice with a lentiviral reporter expression vector under control of the Dectin-2 promotor resulted in reporter expression in LC as well as macrophages [62]. In a comparative study including the aforementioned gene promoters and a promoter fragment of the dendritic cell-specific intercellular adhesion molecule-3-grabbing non-integrin (DC-SIGN) gene which encodes for a CLR predominantly expressed by DC and macrophages [59], the DC-STAMP promoter was identified as most potent to induce antigen-specific T cell responses in vitro after transfection of DC [63].

The highly conserved actin-bundling protein Fascin-1 is highly expressed in neuronal cells, and mediates the structural integrity of axons [64]. We have shown that, besides neuronal cells, Fascin-1 is only expressed in activated DC of mouse and human [65]. The Fascin-1 promoter yielded strong and specific activity in DC of both species [66,67]. In subsequent studies we demonstrated that transcriptional targeting of DC using DNA vaccines containing the Fascin-1 promoter to drive antigen expression yielded antigen-specific T helper cell (Th)1-biased immune responses, whereas transfection with CMV promoter-driven DNA vaccines resulted in mixed Th1/Th2 responses [68]. Moreover, Fascin-1 and CMV promotor containing DNA vaccines induced CTL at comparable extent [66]. In various mouse models of allergen-induced airway inflammation and type I allergy [69], as well as a mouse model of multiple sclerosis [70] vaccination with plasmids encoding the relevant allergen/antigen under control of the Fascin-1 promoter showed therapeutic efficacy, especially when co-transfecting expression constructs that encode anti-inflammatory cytokines.

Transcriptional targeting of DC is intended to restrict antigen and adjuvant expression to this APC population, and thereby to prevent addressing of tolerance by regulatory cell types like MDSC and TAM. However, transcriptional targeting of DC will also largely exclude expression of (protein) antigen in B cells and thereby may impair the induction of antigen-specific antibodies. Additional B cell addressing may be achieved by employment of cellular promoters that display activity also in this APC population [51].

### 4.2. Antigen

The use of DNA vaccines in infectious diseases or for tumor therapy enables to focus on sequences that encode immunogenic peptides of a given pathogen or tumor-specific proteins, and allows to include antigen-encoding sequences derived from different proteins within one minigene to induce a broader T cell response [71]. Antigens that are expressed by tumor cells, so called tumor-associated antigens (TAA) can be divided into two categories: tumor-specific shared antigens and tumor-specific unique antigens [72]. Shared antigens are expressed by different tumors and can also be present in normal cells of different tissues, although at lower amounts. Tumor-specific unique antigens, i.e., neo-antigens, are expressed only by (individual) tumors [73]. Usage of shared antigens is more convenient since the sequence of most proteins is well known and a genetic analysis of the mutanome is not needed to select antigen-encoding sequences [74]. However, a major risk of using shared TAA is the induction of autoimmune responses as the immune system will also be turned against healthy tissues expressing the antigen [75]. Neo-antigens seem to be the first choice if designing a DNA vaccine as studies have shown that effector T cell responses are more potent against mutated antigens [76]. Furthermore, antigens with a higher half-life have been shown to induce stronger cytotoxic T cell responses and thereby increased immunogenicity [77].

Codon optimization is mostly needed if the target antigen is of non-human origin, and can strongly enhance antigen expression [62]. Besides, if immunogenic peptides of an antigen are known, and induction of antibodies specific for the target protein are not an issue, only sequences encoding relevant T cell antigen epitopes may be included [78]. Even if the encoded antigen is expressed at high amounts, antigen presentation/recognition still may be an obstacle to prompt a sufficient immune response. This problem is addressed by introducing epitope-specific changes in the antigen to increase MHC affinity [79]. Similar efforts are being made to increase the affinity of the MHC/peptide complex to T cell receptors [80]. The latest approach to increase antigen presentation is the use of xenogenic antigens [81]. This approach was very successful in the development of the USDA-approved DNA vaccine against canine melanoma.

In current studies, both the strategy to administer a DNA vaccine which encodes for a full length protein as a source of antigen and the administration of a DNA vaccine which encodes for different peptide antigens are followed. While the former approach aims to induce a cellular and concomitant humoral immune response, the latter focuses on the induction of T cell responses at the expense of antibody production. The design of an antigen-encoding expression unit includes the selection of several immunogenic antigens derived from one or different proteins to be presented via MHCI/II to yield parallel CD4^+^/CD8^+^ T cell activation, conceivably codon optimization, and eventually strategies to boost MHCII antigen presentation, e.g., by introducing of the invariant chain.

### 4.3. Adjuvant

To avoid induction of vaccine-mediated antigen-specific tolerance, APC need to be activated by a so-called danger signal which promotes the upregulation of antigen presenting receptors, costimulators and pro-inflammatory mediators for efficient T cell activation and polarization [13]. To this end, antigen-encoding vaccines have been coadministered with established adjuvants like aluminium salt-based Alum [82] or immunostimulatory CpG oligonucleotides which engage the endosomal located toll-like receptor (TLR)9 to stimulate APC [83]. Intradermal application of plasmids was observed to yield Th1-biased immune responses due a CpG-rich element located within the ampicillin resistance gene [84]. Besides TLR9, cytosolic DNA sensors that mediate activation of the stimulator of interferon genes signaling pathway contribute to the intrinsic adjuvant effect of DNA vaccines [85].

Apart from the intrinsic adjuvancy of plasmid DNA, codelivery of plasmids that encode transcription factors as genetic adjuvants has been tested to stimulate APC. Upregulated activation of transcription factors of the nuclear factor ‘kappa-light-chain-enhancer’ of activated B-cells (NF-κB) [86] and the interferon-regulatory factor (IRF) [87] family are hallmarks of APC stimulation by danger signals. Shedlock and coworkers have demonstrated that codelivery of a HIV protein encoding DNA vaccine and of an NF-κB p65 expression construct by in vivo electroporation of mice elevated both cellular and humoral immune responses [88]. Codelivery of an influenza antigen encoding DNA vaccine and an IRF-3 encoding vector by biolistic transfection of mice resulted in an increase of activated CD4^+^ and CD8^+^ T cells [89]. Cotransfection with an IRF-1 expression vector yielded enhanced viral antigen-specific antibody production, whereas enhanced expression of IRF-7 increased both T cell and B cell responses. However, in combination with a HIV tat antigen DNA vaccine delivered by intramuscular injection, only codelivery of an IRF-1 encoding vector elevated the intended CTL/Th1 response [90]. To achieve coactivation of IRF and NF-κB transcription factors, Luc and co-workers immunized mice with an influenza protein encoding DNA vaccine and a plasmid encoding the virus-induced signaling adaptor protein which resulted in elevated anti-viral T cell responses [91]. In a comparative study, Larsen and colleagues assessed the ability of codelivered expression vectors for numerous different TLR signaling adaptors to enhance immune responses [92]. Of more than seventeen different signaling adaptors tested, only combined coadministration of Tak1 and Tram expression vectors enhanced CLT responses in vivo.

The aforementioned strategies aim to enhance the activation state of transfected APC. In other approaches DNA vaccines were coadministered with plasmids that encode either costimulatory receptors in transfected APC to enhance their T cell stimulatory capacity as reported for CD80 and CD86 used e.g., by de Andrés and colleagues to evoke potent anti-Visna/Maedi virus T cell and antibody responses in immunized sheep [93]. More often, DNA vaccines were codelivered with expression constructs for soluble mediators like cytokines or chemokines that exert stimulatory/chemoattracting effects on APC and other immune cells [13]. For example, IL-12 is produced by activated APC to promote Th1 differentiation, but also stimulates APC themselves [94]. Accordingly, administration of an IL-12 expression construct in combination with a multigene HIV DNA vaccine by intramuscular electroporation resulted in enhanced antigen-specific CD4^+^ and CD8^+^ activation in healthy volunteers [95]. IL-15 is also released by activated DC, and exerts broad stimulatory effects on effector CD4^+^ and CD8^+^ T cells, B cells, NK cells as well as DC [96]. In several studies a pronounced effect of cotransfected IL-15 was observed as reported e.g., by Sun and coworkers demonstrating that a fusion construct of a Mycobacterium antigen and IL-15 applied intramuscularly into mice yielded pronounced NK activity, an enhanced Th1 and CTL response as well as elevated antibody titers [97]. Altogether, while different cytokines were employed as genetic adjuvants to stimulate both APC and other types of immune cells [45], IL-2 acts mainly on T cells (and NK cells) [98]. Hence, its overexpression is intended to support sustained activation of exhausted T effector cells and to overcome its depletion by Treg [99].

More recently, as an alternative to promote APC and T cell activation by (over)expression of intracellular or secreted factors, the concept of regulating cell activation by RNA interference has gained attention [100]. For this, either specific silencer RNA or plasmids that encode short hairpin RNA which specifically target intracellular mRNA encoding inhibitory factors are applied. By this approach transcription factors like signal transducer and activators of transcription 3 which is known to induce expression of protolerogenic factors [101] or their downstream targets like the coinhibitory surface receptor PD-L1 [102] that counteract APC-mediated costimulation are targeted. Furthermore, micro-RNA (miRNA) species which regulate the activation state of APC have been recognized as interesting targets [103]. For example, delivery of a plasmid harboring multiple miRNA consensus bindings sites, termed miRNA sponge, is intended to limit their inhibitory effect on activation-associated mRNA targets [104].

In current human DNA vaccination approaches antigen-encoding DNA vaccines are coadministered with soluble immunostimulatory agents which often have been approved for anti-tumor adjuvant therapy [105]. Besides, expression vectors that encode cytokines [106] or chemokines [107] are clinically tested. So far, DNA vaccines that integrate both the antigen expression unit and a molecular adjuvant into a single plasmid have been tested in a limited number of studies only.

### 4.4. Vector Backbone

For transfection the prokaryotic part of a DNA vaccine is not necessary, and early studies have shown that the overall size of a DNA vaccine inversely correlated with transfection efficiency even in mitotically active cell lines [108]. This effect was attributed at least in part to silencing of transgene expression as a consequence of heterochromatin formation of bacterial components spreading into the promoter/vaccine expression cassette [109]. To circumvent this problem, the concept to delete prokaryotic sequences of the plasmid after its propagation in bacteria was developed, yielding so-called minicircle DNA [110]. More recently, Lu and colleagues reported that inclusion of A/T-rich sequences into the vector backbone prevented transcriptional silencing [111]. However, in a growing number of studies, increased efficacy of minicircle DNA vaccines as compared with conventional plasmids has been demonstrated [112].

Concerning the combined administration of DNA vaccines and genetic adjuvants (see above), bicistronic vectors are available which enable expression of both (or more) genes in cis [113]. Such vectors contain either two different promoters to independently express both transgenes or both expression units are separated by sequences like an internal ribosomal entry site (IRES) to mediate cap-independent translation of the downstream expression unit [114]. Especially due to the frequent observation of strongly different expression intensities of both transgenes, as an alternative a virus-derived T2A sequence was introduced instead [115]. After translation, this peptide sequence is recognized by an endogenous protease which mediates posttranslational cleavage of the different transgenes [116]. Usage of an IRES or T2A sequence is interesting when using a cell type-specific promoter for transcriptional targeting of APC.

Efficient transfer of DNA vaccines into the cell nucleus for subsequent expression is an important obstacle for success of transfection, especially in case of mitotically inactive cells like APC [117]. Quite early, certain DNA sequences were identified that mediated nuclear translocation. One of the first identified was the simian virus (SV)40 enhancer region, and transcription factors binding this site in the cytoplasm facilitated active nuclear entry of plasmid DNA due to their nuclear localization signal (NLS) [118]. As a consequence, in some approaches the SV40 enhancer site was included into the vector backbone [11]. Alternatively, the NLS activity of virus-derived peptides like the SV40 large T-antigen was exploited to facilitate nuclear plasmid translocation by attaching these peptides either directly to the vector backbone [119] or to DNA delivery systems [120].

## 5. Nano-Carriers for Transfer of DNA Vaccines

Nano-carriers (NC) offer the advantage to shield the DNA vaccine from degradation by DNases and other enzymes [121]. Surface modifications of NC with moieties like antibodies or natural ligands like carbohydrates may enable direct targeting of DC [16]. As outlined above, direct transfection of APC would prevent unwanted uptake of antigen and adjuvant by MDSC and TAM that promote tumor tolerance [122]. In addition, internal expression of the transgene ensures that sufficient amounts of antigen are presented via MHCI to yield robust CD8^+^ T cell responses [16].

NC are defined as particles of 1–1000 nm in size with an interfacial layer that can be composed of different materials [123]. They have gained scientific interest due to their unique properties and the wide range of applications linked to the variance in composition, size, shape and surface modifications [124]. So far, NC are used predominantly as delivery systems for drugs, adjuvants or nucleic acid-based vaccines contributing to the emerging field of nano-vaccines [125]. One of the most frequent applications of NC is the use in tumor therapy [126] as they have many advantages over classical chemotherapeutics and other pharmacology agents like improved bioavailability [127], organ- [128] or cell type- [16] specific targeting, tunable drug release [129], and addressing of organelles [130]. The exact mode of interaction of NC with a cell membrane is strongly influenced by the size, charge, shape, and hydrophilic/hydrophobic surface properties of the former, and uptake most often occurs either by membrane penetration which is associated with transient appearance of holes or by receptor-mediated endocytosis [131]. Both cellular uptake and endosomal release of NC-complexed DNA is enhanced by cell penetrating peptides (CPP) which are either attached directly to DNA [132] or to the DNA-complexing NC [133]. However, most of these studies have been performed under serum-poor conditions in vitro, and the possibility of rapid formation of a protein corona around the NC and engagement of negatively charged serum components by CPP prior to cellular engagement needs to be taken into account [134]. NC surface modifications like dense decoration with polyethylene glycol (PEG) have been introduced to limit serum protein adsorption, and to retain CPP activity [135]. Major safety concerns associated with the use of NC are are biocompatibility, biodistributiion and clearance, and the induction of unwanted immune reactions [136]. For example, repetitive application of PEG-coated NC may result in the arisal of PEG-specific antibodies [137]. The development of new NC-based delivery systems stirred up hope in the field of gene therapy for cancer treatment [138]. APC are assigned to recognize pathogens, so particles that are of similar size as pathogens should be easily engulfed [139]. While DC normally ingest particles that are virus-like in size (20–200 nm) [140], macrophages usually engulf larger particles (0.5–5 µm) [141], but for NC the efficacy of uptake by APC is strongly dependent on the surface properties and shape of the NC [142]. Cationic particles are internalized better by APC, but are also more likely to induce platelet aggregation and hemolysis [143]. Also the shape of the particle is crucial for uptake. For gold NP (AuNP) it was demonstrated that spherical particles are more efficient than any other shape [144]. Additionally, hydrophobicity, surface modification and the delivered cargo can influence the interaction with immune cells [145]. It needs to be considered that all these factors also influence the interaction with serum proteins, i.e., that modifications can have unpredictable effects in the organism [121].

As outlined below, the field of nanomaterials is still emerging resulting in new designs that open new opportunities for use like the development of virus-like particles (VLP) [146] and magnetic particles which may be directed in vivo by applying magnetic fields [147], and the introduction of tumor microenvironment-triggered drug release mechanisms [148]. Still, there are many challenges linked to the use of NC including the issue whether active targeting to specific cells and tissues is possible and needed for therapeutic efficacy [149].

NC-mediated delivery of nucleic acid-based therapeutics is a very promising approach, but several extra- and intracellular barriers still limit transfection efficacy [150] Hence, NC intended for the delivery of nucleic acid-based therapeutics need to fulfill a number of requirements:

(i) The delivery vector has to offer a sufficient capacity to efficiently package DNA/RNA per se, which is an obstacle especially for longer plasmid DNA [151], in order to enable delivery of a sufficient amount of molecules per target cell [152].
(ii) The delivery system has to show stability against serum proteins that may form a protein corona around the NC and thereby affect its targeting and uptake efficiency [134].
(iii) After uptake by the cell, the NC cargo has to evade endo/lysosomal degradation and to enter the cytoplasm by endosomal escape [153]. While released mRNA is translated directly in the cytoplasm, DNA vaccines need to translocate into the nucleus for subsequent transcription which may be enhanced by NLS [154].

### 5.1. Nano-Carriers Composed of Inorganic Material

There are numerous different types of NC regarding material, size and shape, each having advantages and disadvantages in the field of nanomedicine. Inorganic nanomaterials have a rigid structure and a controllable synthesis allowing simple modification.

AuNP showed low cytotoxic effects, were biocompatible and have been used as carriers for virus-derived antigens [155] as well as DNA vaccines since plasmid DNA can be complexed directly on the surface of the particle [156]. In addition, protein-coated AuNP were reported to possess intrinsic immunostimulatory capacity as they mediated DC activation [157]. The transfection efficiency was increased by the use of CPP to coat the AuNPs, thus minimizing influences from the cell environment on the plasmid DNA and facilitating cell uptake [158].

Another interesting candidate for nucleic acid delivery are silica-based NC. These are biocompatible, and can deliver different types of cargo [159]. Of note, such NC were demonstrated to passively target tumor tissues [160]. Mesoporous silica NC have been used for DNA transfection of cell lines in vitro [161]. In general, such NC allow to control the release of cargo by modification of pore size and shape as well as surface functionalization [162].

Graphene oxide is a compound consisting mainly of carbon and oxygen with a layer structure. Because of the electrostatic π-π-stacking interactions it shows a high loading capacity as well as a controlled release of cargo [163]. Additionally, graphene oxide protects nucleic acids from cleavage, making it a very remarkable candidate for gene therapy [164]. For negatively charged carbon NC direct transfer of DNA via the cell membrane into the cytosol was demonstrated [165]. Another type of carbon-based nanomaterial suitable for transfer of nucleic acids are carbon nanotubes (CNT). CNT are small and chemically inert. However, their hydrophobicity limits their use in biomedical application as CNT are poorly soluble in water [166]. To improve their biocompatibility, CNT can be modified covalently, but this often reduces the loading capacity of nucleic acids as well as the intracellular release of the cargo [167].

Magnetic particles like iron oxide core particles have the unique ability not only to provide cargo delivery but also allow detection in vivo by magnetic resonance imaging [168]. An approach tested in the clinic for cancer therapy is to target magnetic particles to the tumor tissue and then to induce magnetic hyperthermia [169]. To improve biocompatibility of magnetic particles, they are often coated with proteins, polymers or polysaccharides, which reduces cytotoxic effects and can improve their DNA transfecting properties [170].

### 5.2. Lipid-Based Nano-Carriers

Lipids can also be used for gene delivery. Felgner and colleagues were the first to demonstrate that *N*-[1-(2,3-Dioleoyloxy)propyl]-*N*,*N*,*N*-trimethylammonium chloride (DOTAP) can transfer DNA into cells [171]. Complexes composed of DOTAP and protamine-condensed DNA showed good transfection efficiencies in vivo [172]. Cationic lipids are generally very suitable for transfection as they preferably interact with the anionic cell membrane thus facilitating uptake of the transfection complex into the cell [173]. Charge-driven interaction with cellular receptors initiating endocytosis was shown as the uptake mechanism for cationic liposomes [174]. Furthermore, cationic lipids show great binding of DNA and the forming liposomes protect their cargo from degradation. A positive effect is achieved by the addition of a neutral helper lipid, which stabilizes the lipid-bilayer and may promote endosomal escape of passenger DNA either due to pH buffering of protonatable groups or after fusion with the endosomal membrane [175]. In case of cationic DOTAP-based liposomes containing cholesterol as a helper lipid, macropinocytose was identified as the major uptake mechanism [176]. More recently, incorporation of coiled-coil lipopeptides into the liposomal bilayer was demonstrated to promote interaction with cell membrane receptors and direct release of cargo into the cytosol [177]. Liposomes also can encapsulate protein antigens and are commonly used as adjuvant delivery systems as they spontaneously rearrange into nanostructures [178]. Several liposomal protein-based substances haven been approved decades ago for prophylactic vaccination against herpes simplex virus A [179] and influenza [180].

### 5.3. Protein-Based Nano-Carriers

Proteins constitute a favorable material for NC generation since they are biocompatible, biodegradable and typically show low cytotoxicity [181]. A good choice for a gene delivery system is gelatin B combined with protamine sulfate [182]. The overall negative charge of the protein switches in the endosome leading to a release of cargo. The major drawback of this system is the relatively low DNA loading capacity. Endogenous proteins can be also used to design NC for gene delivery. A good example is albumin which is generated largely by the liver and constitutes the most abundant serum protein [183]. Albumin has been demonstrated to exert several tasks via transient binding of other extracellular components, including the protection of proteins and fatty acids from peroxidative modifications [184], ion transport, and adsorption of drugs which affects their bioavailability. In line, albumin has many reactive groups on the surface allowing chemical modifications [185]. NC consisting of an albumin core and chitosan as an outer layer successfully transfected different cell lines [186], and when surface-modified with ethylenediamin for introduction of cationic properties served to deliver siRNA into metastatic tumor in vivo [187].

VLP which consist of various self-assembling viral proteins may constitute suitable candidates for gene delivery [188], as they combine the ability of the virus to interact with immune cells while lacking infectious properties [146]. However, VPL proteins were demonstrated to serve as antigens due to their exogenous origin which constitutes an unwanted side effect [189]. Anyway, the first VLP-based vaccine was commercialized in 1986, and by now several anti-viral and one malaria-directed vaccine are clinically used, most often in combination with the classical adjuvant Alum [190].

### 5.4. Polymeric Nano-Carriers

Polymer-based nanomaterials have gained interest because of their electrostatic interactions with nucleic acids resulting in protection from enzymatic degradation [191]. Furthermore, cationic polymeric nanomaterials are cheap, non-immunogenic, safe and have a greater DNA-loading capacity than viruses. The first polymeric particle reported to enhance transfection was diethylamino-dextran in the late 1960’s [192]. Polymeric systems are often surface-modified to prolong their circulation time in the bloodstream to enable more specific or even cell-targeted delivery. One option to shield NC from the formation of a protein corona determining cellular interactions is the use of PEG [193]. Cationic polylactides are an example of this problem and it was shown that PEGylated particles performed better in serum at high polymer/DNA ratios than commercially available transfection reagents [194]. The importance of polymer-based materials was emphasized when it was early demonstrated that cationic polymers not only bind to DNA but are also very effective in condensing it to form structures resembling viruses [195]. Among the best studied polymeric nanomaterials are poly-d,l-lactide-co-Glycolide (PLG) [196] and poly-d,l-lactic-co-Glycolic acid (PLGA) [197]. PLG- and PLGA-based particles are biocompatible, biodegradable and show a sustained release of a wide range of different cargo molecules [198], including DNA [199]. Poly-L-lysine (PLL) is a natural polymer with a peptidic structure and therefore highly biodegradable. However, the transfection efficiency of PLL particles was lower than obtained using polyethylenimine (PEI) [200]. Polymeric structures achieved a milestone in 1988 as the first receptor targeted transfections were performed. For this, asialoglycoprotein was introduced into PLL targeting its receptor which resulted in increased transfection in vivo [201]. To improve the transfection efficiencies of polymeric systems equipped with targeting moieties, in early approaches inactivated adeno- [202] and rhino- [203] viruses were used in combination with receptor targeting polyplexes. To enhance endosomal escape of the systems, proteins have often been used. In this regard, a sulfhydryl-activatable listeriolysin O/protamine conjugate showed enhanced delivery of DNA into the cytosol [204]. As an alternative, polyamidoamine dendrimers were shown to exert a prominent proton sponge effect as the amines are only slightly protonated at neutral pH [205]. PEI was reported to yield a better transfection capacity as a linear compared to a branched polymer which suggested that unpacking of the DNA is more complicated when complexed with the latter [206]. However, PEI is not biodegradable and shows high toxic effects in a molecular weight-dependent manner [207]. Additionally, cationic polymers in general can be recognized by the immune system and were shown to trigger complement activation, which can lead to an untimely clearance of the NC [208]. In other approaches, the suitability of natural polymers to generate NC has been demonstrated. For example, chitosan was shown to constitute a suitable DNA carrier [209], and bears intrinsic adjuvant activity [210], similar to other polysaccharide-based polymers like inulin [211] and others [212].

## 6. Route of Vaccination

**Figure 3:**
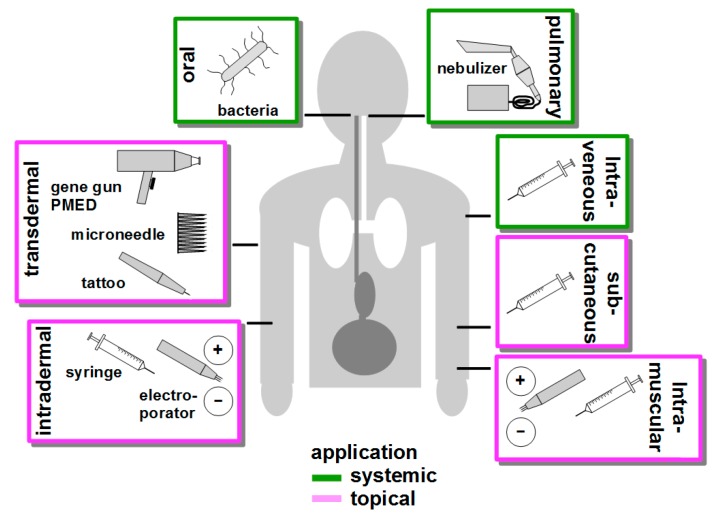
Routes of DNA vaccine delikvery. DNA vaccines may be delivered systemically by intraveneous injection to reach secondary lymphatic organs, by oral application of (attenuated) bacteria as a vehicle to confer uptake of DNA by intestinal APC, and by pulmonary administration of nebulized DNA to achive uptake by lung cells. Transdermal delivery primarily adresses LC, and both needle-free delivery of particle-adsorbed DNA vaccines by helium pressure (gene gun, PMED) and needle-based administration via microneedles and tattoo devices are clinically tested. Transfection of cutaneous APC as well as of non-APC by intradermally injected DNA vaccines is enhanced by immediate electroporation. Subcutaneous injection mainly results in transfection of fibroblasts and keratinocyts, which express transgenes and release antigen for uptake APC. Likewise, intramuscular injection of DNA vaccines primarily yields transfection of myocytes that express/release antigen for APC uptake, and myocyte transfection rates are enhanced by electroportion at the site of injection as well.

To transfect as many APCs as possible, targeting DNA vaccine delivery to secondary lymphoid organs via systemic application like intravenous injection [213], oral [214] or pulmonary [215] administration is a suitable strategy. Alternatively, DNA vaccines are often applied often topically via the skin [216] or intramuscularly [217]. Figure 3 presents an overview over common DNA vaccination routes.

Intraveneous application of DNA vaccines inprinciple allows to reach APC located in secodnary lymphoid organs. However, the physicochemical characteristics of DNA-complexing NC determine the biodistribution of the DNA vaccine. For example, PEI-complexed reporter expression vectors preferentially transfected lung cells [218], whereas lipsomomal formulations transfected liver cells [219]. In general, numerous types of NC were demonstrated to interact with blood components which thereby form a protein corona around the NC which in turn may strongly affect its cellular binding properties [134]. For example, we have recently shown that NC coated wtih dextran to confer biocompatibility triggered the lectin-dependent pathway of complement activation which resulted in deposition of C3 fragments on the NC surface, and preferential binding to splenic B cells via their complement receptor [220].

For oral administration of DNA, non-pathogenic (*L. lactis* [221] or attenuated (*S. thyphimurium* [222] and *L. monocytogenes* [223]) bacterial strains are often used as vectors, termed bactofection [224]. In the intestine, these bacteria may be phagocytosed directly by mucosal DC/macrophages spreading extensions into the gut lumen or after M cell-mediated transcytosis at the Peyer’s patches [225]. After phagocytosis, plasmid DNA is released from phagolysosome, and the numerous bacteria-associated danger signals result in profound APC activation [226]. More recently, ‘bacterial ghosts’ which constitute only the bacterial envelope have been introduced as carriers for DNA vaccines [227]. Aside oral application, bacteria were shown to confer transfection of APC when applied at other mucosal sites, e.g., when applied intranasally [228]. More recently, the DNA vaccine delivery properties of orally applied bacteria, for example with regard to phagolysosomal escape have been improved by additional coating with NC [229].

Compared with other sytemic routes of DNA vaccination, pulmonary application of aerolized DNA vaccines is a rather new approach [230]. Usage of naked and NC-complexed DNA was demonstrated to yield transfection of lung epithelial cells [231]. Hence, so far research focusses on therapeutic treatment of local gene defects as in case of cystic fibrosis [232].

The skin constitutes an interesting target organ for DNA vaccination due to the rather high frequency of cutaneous DC. For example, human skin, depending on the specific site, contains 200–1000 LC per square millimeter [233]. Furthermore, in skin (activated) DC are the only cell populations showing migratory behavior towards draining lymph nodes to evoke T cell responses [234]. Different transdermakl DNA vaccination strategies have been developed, and their suitability is clinically tested [235]. Needle-free biolistic transfection as mediated by gene gun [69] and PMED (particle-mediated epidermal delivery [236] devices transfers microparticle-adsorbed DNA into the epidermal layer by helium force to transfect LC, dermal DC (and keratinocytes). Of note, the physical stress associated with biolistic transfection, was reported as sufficient to mediate activation and emigration of directly transfected DC [237]. Microneedles which are produced from various materials and techniques display lenghts below one micron [238] and tattooing devices [239] address these cell types as well. Conventional intradermal administration of DNA vaccines by syringes aims to transfect dermal DC (and fibroblasts). Based on the observation that after intradermal injection of DNA a short electrical pulse, termed electroporation, mediates several-fold enhanced transfection has resulted in the development of a number of according devices tested in clinical studies [240]. Similarly, in vaccination studies transfection rates of myocytes after intramuscular injection of DNA, intended to generate antigen for uptake by APC, were found strongly elevated by electroporation as well [106]. In general, electroporation in the context of transdermal [241] and intramuscular [242] DNA vaccination was reported to result in local activation of innate immunity which may be a consequence of e.g., electroporation-induced cellular stress reactions, including necrosis.

Concerning the success of immunization of different DNA vaccination routes, the recent phase I trial CUTHIVEC which assessed in a comparative manner the efficacy of different DNA vaccine administration routes showed increased antigen specific CD4^+^ and CD8^+^ responses after combined intramuscular and transcutaneous injection compared to intramuscular plus intradermal injections [43]. The former approach was even more efficient than intramuscular administration followed by electroporation (EP) at the injection side. EP is frequently used to increase the overall transfection efficiency at the injection site and was shown to efficiently enhance immune responses in rhesus macaques after DNA vaccination [243]. In general, the application method itself beyond mediating APC activation may also influence T cell polarization. In a comparative study, intramuscular DNA vaccination resulted in a Th1-biased T cell response (see above), whereas biolistic transfection yielded a Th2 response [244].

With regard to the distribution of DNA vaccines complexed with NC it is noteworthy that small particles are easily transported into the lymph node, while larger particles remain longer at the site of administration [245]. In addition, the route of administration can also account for the fate of the delivery systems. After subcutaneous injection small PEGylated liposomes were found in larger amount in the lymph node than after intravenous or intraperitoneal injection [246]. Concerning NC clearance from the body, NC that are smaller than 8 nm are cleared renally [247], and the extent of renal clearance was shown to correlate with the extent of negative charge [248]. Biliary clearance was observed especially for particles over 200 nm and for strongly charged particles [249].

## 7. Targeting of Antigen Presenting Cells

Conventional application routes of naked and NC-complexed DNA may result predominantly in transfection of non-APC which in turn may generate and release antigen that may be engulfed by APC [14] [8]. However, only DC populations with cross-presenting potential are able to shuttle a fraction of extracellular antigen towards MHCI which enables stimulation of CD8^+^ T cells [16]. Therefore, pronounced CTL activation requires direct transfection of APC [10]. Due to their pathogen-like appearance in terms of size and shape, NC-complexed DNA vaccines may passively target APC like (conventional) DC and macrophages since these cell types are specialized in the uptake of ‘foreign’ material [250]. Usage of either natural ligands or antibodies specific for endocytically active receptors strongly expressed on APC may enable cell type-focussed targeting [251]. Such moieties may be coupled to naked DNA or DNA complexing NC.

For targeting, CLR that are predominantly expressed by DC and macrophages constitute suitable candidates [252]. For example, DC-SIGN [253] and the mannose receptor [254] are expressed by either cell type at differential intensity, respectively, and mediate internalization of mannosylated particles. Qiao and coworkers demonstrated that vaccination of mice with a mannosylated cationic liposome that complexed HIV protein-encoding plasmid DNA resulted in improved immune responses in mice [255]. Intramuscular application of a DNA vaccine encoding a botulinum neurotoxin fused with a single chain antibody fragment (scFv) specific for the CLR DEC-205 resulted in improved cellular and humoral immune responses, and protected vaccinated animals from botulinus infection [256]. Fusion of a tumor antigen encoding DNA vaccine with a CD11c-specific scFv was protective in a mouse breast cancer model and slowed tumor growth in a therapeutic setting [257].

## 8. Concluding Remarks

The immunogenicity of DNA vaccines in human ist still to low to yield therapeutically convincing results. However, in the last 15 years different approaches have shown that optimization of different parameters contributes to enhanced transfection and hence immunogenicity of DNA vaccines also in human. Hence, an ideal DNA vaccine will need to be complexed with an APC-targeting NC to prevent extracellular degradation and to enable direct APC transfection, and will contain a NLS to facilitate nuclear entry. The DNA vaccine should contain a promoter that facilitates transcriptional targeting of APC to prevent unwanted antigen expression in tolerance-promoting cell types like MDSC. Furthermore, the DNA vaccine should include a genetic adjuvant which promotes activation of the transfected APC to prevent antigen-specific tolerance induction. Of note, several types of NC bear intrinsic immunostimulatory activity, and the mode of DNA delivery may also result in local inflammation. Both factors need to be considered since they contribute to shape the character of the induced immune response, especially with regard to T cell polarization [258]. With regard to tumor therapy, recent progress in cost effective deep sequencing now allows to identify patients-specific tumor antigens [259].

While administration of a DNA vaccine as outlined above aims to induce antigens-specific immune responses, additional application of agents that inhibit immuno-regulatory myeloid cells like MDSC and Treg as well as the tumor itself may have synergistic effects [260]. Besides chemotherapeutics, checkpoint inhibitors that have recently been introduced into clinical therapy are likely candidates for cotreatment [261].

## Abbreviations

APC	antigen presenting cell
AuNP	gold nanoparticle
CLR	C-type lectin receptor
CPP	cell penetrating peptide
CNT	carbon nanotube
CTL	cytotoxic T lymphocyte Cutaneous and Mucosal HIV Vaccination
CUTHIVAC	cutaneous and mucosal HIV vaccination
DC	dendritic cell
DC-SIGN	dendritic cell-specific intercellular adhesion molecule-3-grabbing non-integrin
DC-STAMP	dendrocyte expressed seven transmembrane protein
DOTAP	*N*-[1-(2,3-Dioleoyloxy)propyl]-*N*,*N*,*N*-trimethylammonium chloride
IRF	interferon-regulatory factor
HLA	human leukocyte antigen
LC	Langerhans cell
MDSC	myeloid-derived suppressor cell
MHC	major histocompatibility class
NC	nano-carrier
NK	natural killer cell
miRNA	micro-RNA
NF-κB	nuclear factor ‘kappa-light-chain-enhancer’ of activated B-cells
NLS	nuclear localization signal
PEG	polyethyleneglycol
PEI	polyethylenimine
PLGA	poly-d,l-lactic-coglycolic acid
scFv	single chain antibody fragment
SV40	Simian virus 40
TAA	tumor-associated antigen
TAM	tumor-associated macrophage
Th	T helper cell
TLR	toll-like receptor
Treg	regulatory T cell
VLP	virus-like particle
